# Intraoperative parathyroid hormone assay benefits surgery for primary hyperparathyroidism when preoperative localisation is negative or not performed

**DOI:** 10.1308/rcsann.2024.0051

**Published:** 2024-09-25

**Authors:** D Scott-Coombes, M Stechman, N Patel, R Egan

**Affiliations:** ^1^ University Hospital of Wales, UK; ^2^ Morriston Hospital, UK

**Keywords:** Parathyroidectomy, Parathyroid, Intraoperative PTH, Localisation

## Abstract

**Introduction:**

Parathyroid localisation is now routine before first-time surgery for patients with primary hyperparathyroidism (PHPT). The aim of this study was to investigate the contribution of intraoperative parathyroid hormone (PTH) (ioPTH) in patients in whom localisation was either not undertaken or negative for a tumour.

**Methods:**

This was a retrospective study of patients undergoing first-time parathyroidectomy for PHPT in a regional endocrine centre. Data were collected prospectively (Microsoft Excel) and the all-Wales electronic patient record portal was used to retrieve missing data. Statistical analysis appropriate for nonparametric data was undertaken, with statistical significance reached when *p*<0.05.

**Results:**

Between 1 July 2002 and 31 December 2022, 1,490 patients underwent a first-time parathyroidectomy for PHPT. Of this cohort, 1,133 patients had at least one positive imaging modality; the study group consisted of 343 patients that had negative imaging, and 13 that had no preoperative localisation. Patients with MEN-1 (*n*=26), an incorrect diagnosis (*n*=4), or less than six months follow-up (*n*=6) were excluded. Of the remaining 321, 106 patients underwent surgery without ioPTH (Group A), 215 cases with ioPTH (Group B). In Group B there were more women (170 female/45 male; 79% vs 67 female/37 male; 63% *p*=0.002, chi-squared), lower calcium (median [range] 2.77 [2.63–3.24] mmol/l; vs 2.85 [2.60–4.52] *p*=0.001) and lower PTH (12.0pmol/l [3.4–39.5] vs 14.4 [3.9–97.0] *p*=0.001) and smaller weights of resected tissue (320mg [50–9,000] vs 454 [46–8,280] *p*=0.02) (Student’s *t*-test). The rate of multiple gland disease was similar (Group A 29%; Group B 27%). The rate of normocalcaemia at 6 months was significantly higher when ioPTH was used (Group B 202/215; 94% vs Group A 90/106; 85%) (*p*=0.014, chi-square test). The sensitivity and specificity of ioPTH was 98.5% [confidence interval (CI) 96.2–99.6] and 91.2% [80.7–97.0] (positive predictive value 99.9%, CI 93.6–100.0).

**Conclusion:**

Despite milder hyperparathyroidism and smaller tumour weight, the outcome in patients in whom ioPTH was used was superior, with failure rates 2.5-fold higher in the cohort where ioPTH was not utilised. The results of this study demonstrate that ioPTH is a valuable adjunct for the surgeon in cases where localisation has failed or not been undertaken.

## Introduction

At the onset of the modern era of endocrine surgery, the traditional surgical approach for patients with primary hyperparathyroidism (PHPT) was to perform a bilateral neck exploration, identify all four glands and to excise the abnormal one(s).^1^ The precise identification of all normal glands is required for the unequivocal diagnosis of an abnormal gland and, because localisation studies did not identify normal glands and seldom identified multiple abnormal glands, localisation was considered unnecessary at that time.^2^ Such an approach achieved successful cure rates of over 95% when undertaken by expert surgeons.^3,4^ In its infancy, parathyroid localisation was reserved for finding rare ectopic glands after a first exploration had failed.^5^ During the 1990s, localisation techniques became more accurate and less invasive, with ultrasound and (sesta) multiplex ion beam imaging (MIBI) techniques being most favoured.^6^ Improved localisation outcomes led Russell to pioneer targeted parathyroidectomy guided by the localisation result.^6,7^

Between 1990 and 2000, there was a world-wide paradigm shift from bilateral neck exploration towards unilateral surgery guided by the increasing accuracy of localisation studies.^8^ The drawback of this unilateral approach was that contralateral pathological glands would be missed, with inadequate recognition and treatment of multiple gland disease (MGD) being the most common cause of failure in surgery for PHPT.^9^ Localisation studies are just as unreliable in detecting MGD now as they were in the latter parts of the twentieth century.^10,11^ Following the elucidation of a rapid assay to measure intact parathyroid hormone (PTH),^12^ George Irvin III, a surgeon in Florida, introduced intraoperative measurement of PTH (ioPTH) to guide surgical decision-making regarding unilateral or bilateral neck exploration.^13^ Irvin and his team devised the now familiar Miami criteria to guide the use of ioPTH and to signify satisfactory surgery, based on results from a large case series.

But just three years after his seminal paper, Irvin published a second paper describing a new parathyroidectomy technique that combined the use of localisation and ioPTH assay.^13,14^ In other words, the use of ioPTH was linked intrinsically to successful localisation and, for the past 25 years, most surgeons have adopted the philosophy that ‘localisation tells you where to start and ioPTH tells you when to stop’. Therefore, when localisation is unsuccessful, guidelines recommend bilateral neck exploration and make no mention of ioPTH.^15^

This study aims to test the hypothesis that ioPTH is a useful adjunct to parathyroidectomy for PHPT even in cases where localisation is either not undertaken or when it has failed.

## Methodology

In this study a retrospective cohort of consecutive patients undergoing first-time parathyroidectomy was identified from a prospectively maintained departmental database, and suitable cases were analysed following defined exclusions. Demographic data, intraoperative data, including extent of surgery and ioPTH results, and follow-up calcium and PTH data were recorded. Missing data were obtained from the all-Wales electronic clinical portal in the hospital informatics system. The protocol of parathyroid surgery and ioPTH by our unit has been published previously.^16,17^ When localisation is negative, the left side of the neck is explored first, followed by the right side if no left-sided tumour is found. When a tumour is excised, ioPTH is measured. For patients in whom there was no localisation, a bilateral neck exploration was performed with the intent to identify all four glands.

Categorical data were explored using the chi-square test and continuous variables compared using Student’s *t*-test. Statistical significance was taken at the 95% level (*p*<0.05). Sensitivity, specificity and positive predictive value (PPV) were calculated using cross-tabulation. Ethical approval was deemed unnecessary by the university health board research department, as this was a retrospective service evaluation analysis.

## Results

**Figure 1:**
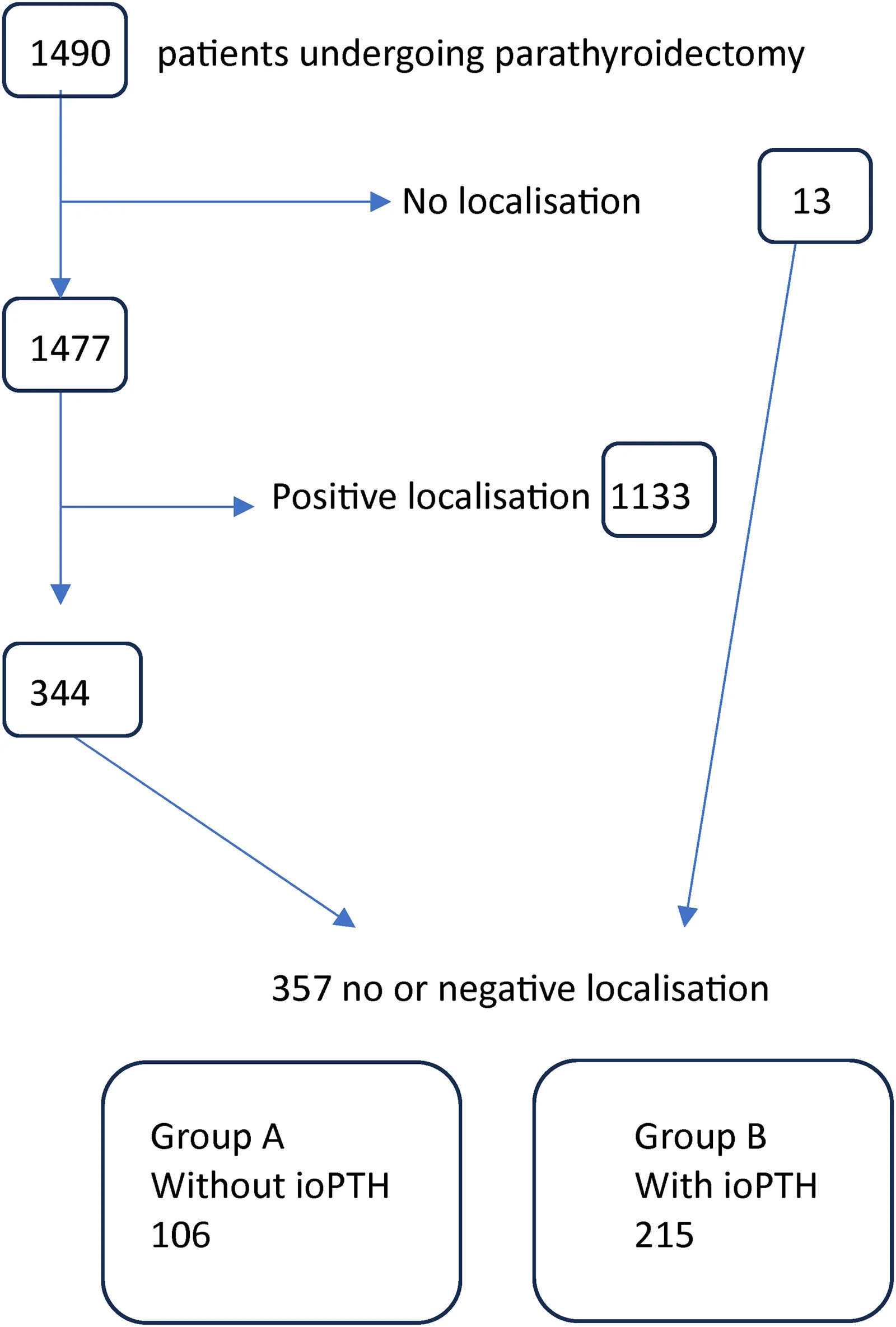
Flow chart of patient recruitment. ioPTH = intraoperative parathyroid hormone.

Between 1 July 2002 and 31 December 2022, 1,490 patients underwent a first-time parathyroidectomy for PHPT in our regional tertiary centre. Of these, 357 patients either had no scan (*n*=64) or both negative ultrasound and negative MIBI scans (*n*=293). Patients in whom the incorrect diagnosis (*n*=4) became apparent, those with MEN-1 (*n*=26) and those with less than six months follow-up (*n*=6) were excluded. Of the remaining 321, 106(33%) patients underwent surgery without ioPTH (Group A) and in 215(67%) cases ioPTH was used (Group B) (see flow chart in Figure 1). In group B there were more women, the degree of hypercalcaemia was milder and the median tumour size was smaller (*p*<0.05) (Table 1).

**Table 1:** Characteristics in each group

	Group A (without ioPTH)	Group B (with ioPTH)
Number of patients	106	215
Women	63% (66 patients)	79% (169 patients)
Median (range) age (years)	64 (12–86)	65 (22–89)
Median (range) calcium (mmol/l)	2.85 (2.60–4.52)	2.77 (2.63–3.24)*
Median (range) PTH (mmol/l)	14.4 (3.91–97)	12 (3.4–39.5)
Median weight of parathyroid (mg)	454 (46–8,280)	320 (50–9,000)*
Median follow-up (months)	12 (6–192)	14m (6–133)

ioPTH = intraoperative PTH; PTH = parathyroid hormone.

*p < 0.05.

**Figure 2:**
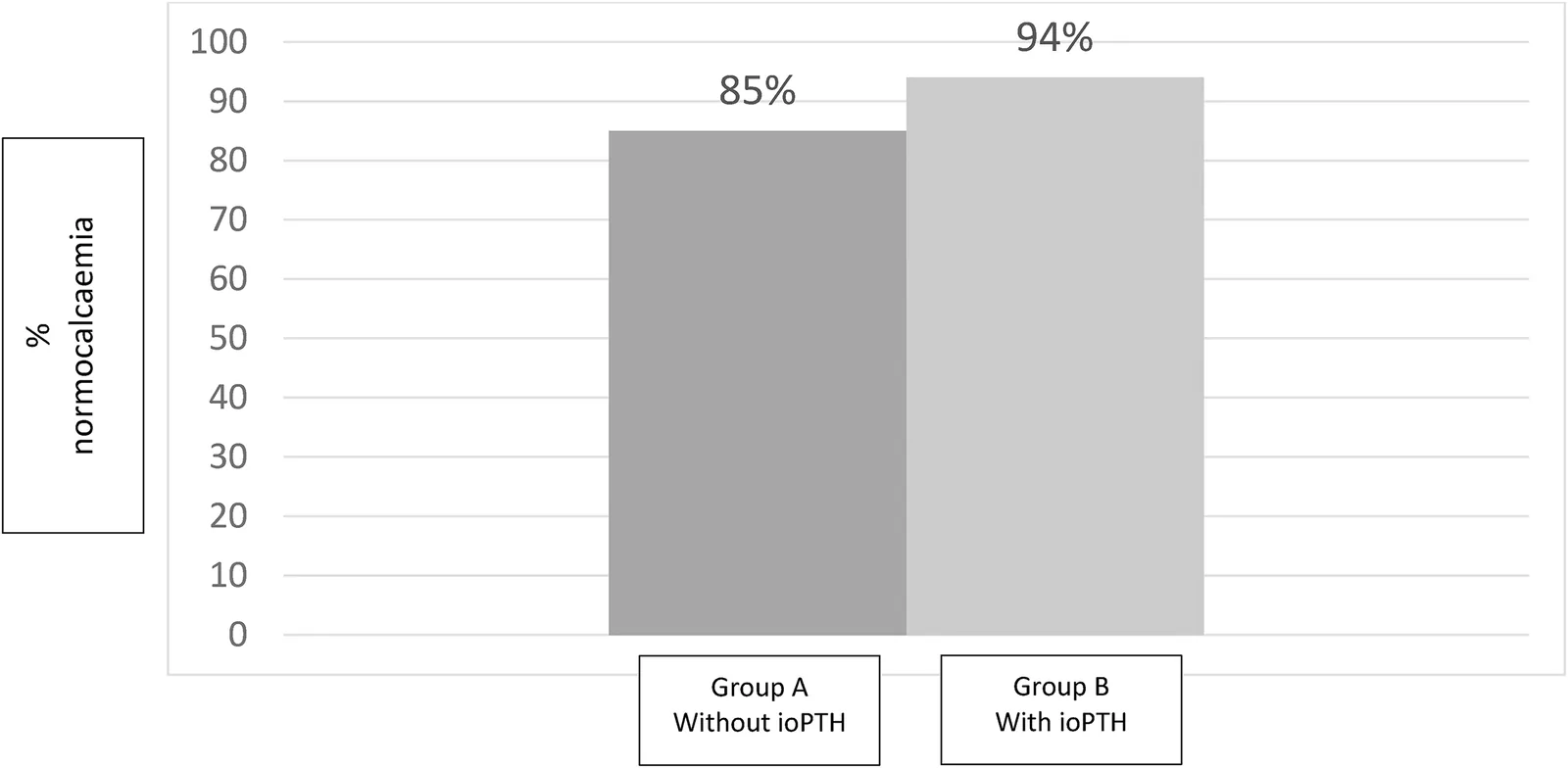
Normocalcaemia rates in group A (without ioPTH) and Group B (with ioPTH). ioPTH = intraoperative PTH; PTH = parathyroid hormone.

In both groups there was a high rate of MGD (Group A 31/106 [29%], Group B 58/215 [27%]). The median (range) cohort follow-up was 14 (6–192) months. At six-month follow-up, there was a statistically higher rate of normocalcaemia in Group B (94% vs 85%) (Figure 2). Regarding the accuracy of ioPTH, the sensitivity was 98.5% (confidence interval [CI]: 96.2–99.6) and the specificity was 91.2% (CI: 80.7–97.0) (Table 2). The PPV was 99.9% (CI: 93.6–100.0%). When the operating surgeons thought that the patient had MGD at the time of the operation, there was little difference in normocalcaemia-at-six-months rates between each group, but when the surgeon thought that the patient had single-gland disease, the rate of normocalcaemia was much higher in Group B (Table 3).

**Table 2:** Statistical outcomes for ioPTH assay

Sensitivity	98.5% [CI 96.2–99.6]
Specificity	91.2% [CI 80.7–97.0]
PPV	99.9% [CI 93.6–100.00]

CI = confidence interval; ioPTH = intraoperative parathyroid hormone; PPV = positive predictive value.

**Table 3:** Outcomes for each group influenced by the surgeon's working diagnosis at the time of the neck exploration

Surgeon's diagnosis	Group A (without ioPTH)	Group B (with ioPTH)
	Success rate (normocalcaemia)	
Double adenoma	5/5;100%	16/18; 89%
Multiple (more than two)	9/10; 90%	26/29; 89%
Single gland disease	72/90; 80%	158/168; 94%*

ioPTH = intraoperative parathyroid hormone.

*p < 0.05.

Regarding the failures (*n*=29), defined as hypercalcaemia >2.6mmol/l at six months, the rate of MGD was high (Group A 10/16; 62%, Group B 11/13; 85%) and the weight of the tumours was low, with 65% (*n*=21) of tumours weighing less than 400mg and less than 200mg in 35% (*n*=11)(*p*=0.6).

## Discussion

As a profession, surgeons' enthusiasm for new technology has often outstripped evidence.^18^ The widespread adoption of parathyroid localisation since the 1990s is an example of this enthusiasm, which fuelled NICE guidance to recommend localisation over ioPTH.^19^ We sought to challenge the prevailing attitude that ioPTH is of value only in patients successfully localised to facilitate a targeted operation.

The patients in this cohort are evenly distributed across this study period and all operations have been undertaken by two consultant surgeons who have obviously become more experienced between 2002 and 2022. Our Unit has been using ioPTH since 2004 and we analysed the contribution of ioPTH to the outcome of parathyroid surgery in patients with PHPT in whom localisation had either not been undertaken or was negative; in other words, to test the value of ioPTH independent of localisation scans.

In our practice, when both ultrasound and MIBI scans are negative, we do not request further imaging such as computed tomography/positron emission tomography. There were 64 patients who did not have any scan. Of these, 22 patients had MEN1, 10 had no localisation during the COVID-19 pandemic to limit the number of hospital visits and 32 (2%) underwent urgent surgery. We did not measure ioPTH in one-third of our patients in this study for reasons including (a) before introduction of ioPTH (*n*=32); (b) COVID-19 pandemic (minimising staff numbers in theatre; *n*=42) and (c) ioPTH or biochemist unavailability (*n*=32). We excluded patients with MEN1 (who will inevitably develop recurrence), those lost to follow-up before six months, and four patients in whom the diagnosis of PHPT was incorrect (three familial hypocalciuric hypercalcemia, one macroglobulinemia).

The Achilles heel of parathyroid surgery is the presence of MGD and failure ensues when this is not recognised. The most reliable way to determine whether a patient has single or MGD is to identify all four glands. In our opinion, it is harder to identify normal glands than parathyroid tumours. One benefit of the ioPTH assay is that it liberates the surgeon from having to find all normal glands once an adenoma has been excised if the ioPTH adequately falls.

Analysis of both historic and current papers reveals that, when surgeons set out to identify all four glands, they do so in only 44–75% of operations.^4,20,21^ Irvin had the honesty to admit that looking for normal glands is difficult, especially in obese patients, and that surgeons tire of searching for normal glands once an abnormal gland has been excised.^13^ This lack of perseverance contributes to surgical failure and we speculate that this explains the difference in outcomes between the two groups. All patients without localisation underwent a bilateral neck exploration, but although the number of glands excised was recorded, the number of glands identified was not.

The high rate of MGD in each of our groups is consistent with the known relationship between MGD and double-negative scans.^16^ We consider it important that patients with double-negative scans are appropriately consented before operative intervention, because MGD is associated with a higher failure rate (7–18%).^16,22^ We found ioPTH assay to be highly accurate with a PPV (the likelihood that if the ioPTH falls that the patient is cured) of 99.9%.

Long-term cure rates remained high in the ioPTH group, despite smaller tumours and more mild biochemical disease, with failure to cure 2.5-fold higher in the non-ioPTH group. This demonstrates the second benefit of ioPTH, namely that it alerts the surgeon to the likelihood of MGD in cases where there is residual hyperfunctioning tissue once a gland has been removed. When the ioPTH has not adequately fallen, it is imperative that all four glands are identified. A failure to do so led to a failure to cure two patients in Group B with presumed double adenoma.

In the 29 failed operations, MGD was the commonest pathology and, in both groups, the tumours weighed less than 200mg in 35% of cases. This is consistent with previous studies reporting negative localisation to be associated with smaller tumours and when the first parathyroid tumour identified weighs less than 200mg, the rate of MGD is 40%.^23^

This study has several limitations, which are inherent with all single-centre retrospective studies. It would be desirable to conduct a prospective study comparing ioPTH-alone with localisation, adequately powered to assess the primary outcomes measure. The data are from a single regional Unit and generalisability is limited, with intraoperative decision-making dependent upon the experience of each of our surgeons, and thus may be impacted by subjectivity. The number of glands identified during the operation was not recorded routinely, only the number of glands excised. The strengths of this study include the large number of consecutive patients with complete postoperative follow-up data, and standardised patient operative management by surgeons who regularly submit parathyroid surgery outcomes data to the national registry.

In conclusion, these results affirm our hypothesis that ioPTH is a useful surgical adjunct that is independent of parathyroid localisation. The commonest reason to fail to cure patients with PHPT is the failure to recognise or adequately treat MGD. Localisation is unhelpful in diagnosing MGD. To achieve high success rates, identification of all four glands should remain the gold standard as determined by the pioneers of parathyroid surgery. This is achieved either visually by means of a bilateral neck exploration (possibly augmented with near-infrared autofluorescence)^24^ or virtually with the use of ioPTH, which tells the surgeon about the function of the remaining parathyroid glands without the need for direct visualisation when the ioPTH adequately falls. The results of this study provide a strong argument for surgeons to adopt ioPTH when localisation scans are negative.
